# Protective Effects of Novel Antioxidant Peptide Purified from Alcalase Hydrolysate of Velvet Antler Against Oxidative Stress in Chang Liver Cells In Vitro and in a Zebrafish Model In Vivo

**DOI:** 10.3390/ijms20205187

**Published:** 2019-10-19

**Authors:** Yuling Ding, Seok-Chun Ko, Sang-Ho Moon, Seung-Hong Lee

**Affiliations:** 1 Department of Pharmaceutical Engineering, Soonchunhyang University, Asan 31538, Korea; dingyuling@naver.com; 2 National Marine Bio-Resources and Information Center, National Marine Biodiversity Institute of Korea, Seochun 33662, Korea; scko0527@gmail.com; 3 Division of Food Bioscience, Konkuk University, Chungju 27478, Korea; moon0204@kku.ac.kr

**Keywords:** velvet antler, alcalase hydrolysate, antioxidant peptide, protection ability, oxidative stress

## Abstract

Velvet antler has a long history in traditional medicine. It is also an important healthy ingredient in food as it is rich in protein. However, there has been no report about antioxidant peptides extracted from velvet antler by enzymatic hydrolysis. Thus, the objective of this study was to hydrolyze velvet antler using different commercial proteases (Acalase, Neutrase, trypsin, pepsin, and α-chymotrypsin). Antioxidant activities of different hydrolysates were investigated using peroxyl radical scavenging assay by electron spin resonance spectrometry. Among all enzymatic hydrolysates, Alcalase hydrolysate exhibited the highest peroxyl radical scavenging activity. Alcalase hydrolysate was then purified using ultrafiltration, gel filtration, and reverse-phase high performance liquid chromatography. The purified peptide was identified to be Trp-Asp-Val-Lys (tetrapeptide) with molecular weight of 547.29 Da by Q-TOF ESI mass spectroscopy. This purified peptide exhibited strong scavenging activity against peroxyl radical (IC_50_ value, 0.028 mg/mL). In addition, this tetrapeptide showed significant protection ability against AAPH-induced oxidative stress by inhibiting of reactive oxygen species (ROS) generation in Chang liver cells in vitro and in a zebrafish model in vivo. This research suggests that the tetrapeptide derived from Alcalase-proteolytic hydrolysate of velvet antler are excellent antioxidants and could be effectively applied as functional food ingredients and pharmaceuticals.

## 1. Introduction

Reactive oxygen species (ROS) are chemically reactive species containing oxygen. ROS are normally produced in living organisms during metabolism of oxygen. Under normal conditions in our body, ROS can be effectively eliminated by antioxidant defense systems such as endogenous antioxidant enzymes and non-enzymatic factors [1]. However, overproduction of ROS by various factors can cause oxidative stress and lead to a variety of pathological conditions, including metabolic impairments such as inflammation, aging, cancer, and cardiovascular diseases [2]. Therefore, sufficient amount of antioxidants need to be consumed to prevent or slow down oxidative stress induced by ROS. The amount of synthetic antioxidants used by humans is under strict regulation due to their potential health hazards [3]. Thus, natural antioxidants without side effects or toxicity have attracted great interest.

Food-derived peptides have shown to be potent antioxidants without serious side effects [4]. As such, to discover bioactive peptides from food proteins and to develop the peptides as alternatives to synthesis antioxidants has been considered by many researchers. In addition, the peptides from gastrointestinal digested food proteins may act as potential physiological modulators of metabolism during gastrointestinal digestion [5,6]. Several recent studies have suggested that animal proteins are good sources to produce antioxidant peptides and demonstrated that animal proteins hydrolystes and/or its antioxidant peptides may promote health by decreasing oxidative stress [7,8,9].

Velvet antler is a typical traditional medicine from animal origin that is recognized in the pharmacopeias of Korea, China, and Japan. In has been used as traditional medicine for over 2000 years. It also used as a functional foods or nutraceutical supplement in New Zealand, Canada, and the USA [10]. The reports support that main prominent bioactive components of velvet antler are polypeptides and proteins [11]. The traditional extraction of bioactive components from velvet antler is generally done via simmering in hot water. However, there is some controversy surrounding this approach, due largely to the extremely limited recovery of bioactive components in water extractions. In recent years, enzymatic hydrolysis using commercial proteases has been successfully applied to extraction of numerous biologically active peptides from a wide variety of food proteins and organisms. Recently, several studies have reported that enzymatic hydrolysate extracted from velvet antler using commercial proteases such as Alcalase, Protamex, pepsin, and Neutrase show a variety of biological benefits, including anti-obesity, anti-inflammatory, and antioxidant effects [12,13,14]. Anti-inflammatory peptides derived from velvet antler protein have also been reported [15].

However, to the best of our knowledge, there have been no reports about antioxidant peptides extracted from velvet antler by enzymatic hydrolysis. Therefore, the objective of the present study was to evaluate antioxidant activities of hydrolysates from velvet antler prepared with five commercial proteases (Acalase, Neutrase, trypsin, pepsin, and α-chymotrypsin) and identify amino acid sequences of purified peptides with the strongest antioxidant activity. Protective effects of purified peptides against 2,2′-Azobis(2-amidinopropane) dihydrochloride (AAPH)-induced oxidative stress in Chang liver cells and a zebrafish model were also investigated.

## 2. Results

### 2.1. Preparation of Enzymatic Hydrolysates from Velvet Antler and Their Peroxyl Radical Scavenging Activities

Velvet antler was successfully hydrolyzed with various commercial proteases such as trypsin, pepsin, α-chymotrypsin, Neutrase, and Alcalase to produce potent antioxidant peptides. Yields of velvet antler enzymatic hydrolysates measured by dry weight were observed to be 34.09%, 12.39%, 38.96%, 23.81%, and 29.75% for trypsin, pepsin, α-chymotrypsin, Neutrase, and Alcalase, respectively (Table 1). Antioxidant activities of these hydrolysates against peroxyl radical were examined using an ESR spectrometer. Their scavenging activities are shown in Table 1. Among these hydrolysates, Alcalase-derived hydrolysate possessed the highest peroxyl radical scavenging activity. Although the trypsin hydrolysate also showed a potent peroxyl radical scavenging activity, Alcalase can produce shorter peptide sequences and terminal amino acid sequences responsible for various bioactivities as well as useful for the production of bioactive peptide [16,17,18]. Therefore, Alcalase hydrolysate was selected to identify antioxidant peptide for further studies.

### 2.2. Purification and Identification of Antioxidant Peptide

**Figure 1:**
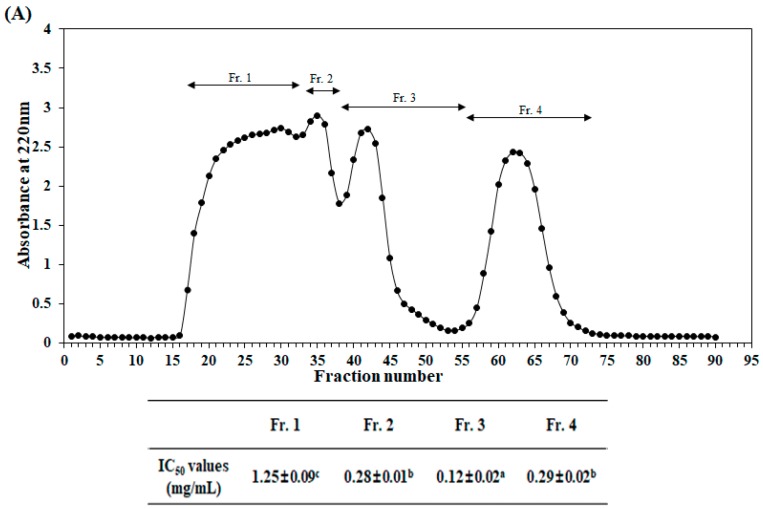

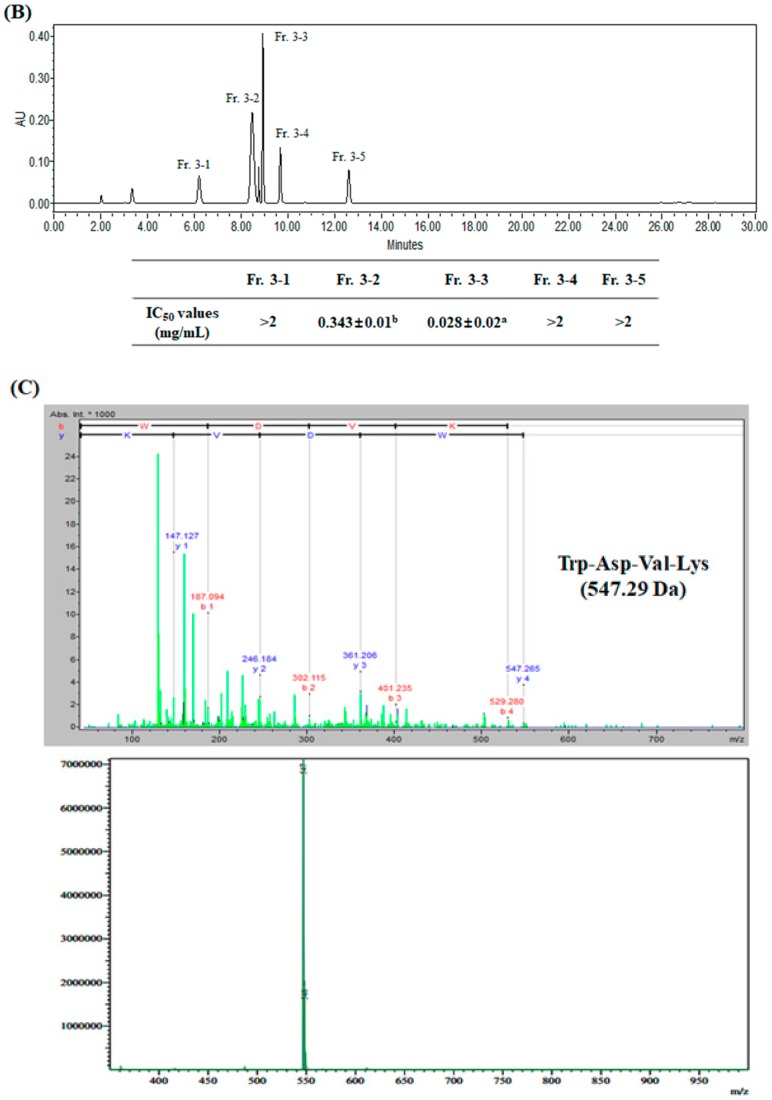
Purification and identification of antioxidant peptide. (**A**) Sephadex G-25 gel filtration chromatogram of <5 kDa fraction from Alcalase hydrolysate (upper panel) and its peroxyl radical scavenging activity (lower panel). (**B**) RP-HPLC chromatogram of the potent peroxyl radical scavenging activity fraction (Fr. 3) isolated from G-25 (upper panel) and its peroxyl radical scavenging activity (lower panel). (**C**) Identification of amino acid sequence and (left panel) molecular weight (right panel) of the purified peptide (TAVL) from Alcalase hydrolysate of velvet antler with a Q-TOF ESI mass spectrometer. These values are expressed as mean ± S.E. from triplicate experiments. ^a–c^ Values with different alphabets are significantly different at *p* < 0.05 as analyzed by Duncan’s multiple range test.

Initially, the Alcalase hydrolysate of velvet antler was cut off by two kinds of ultrafiltration membranes (5 and 10 kDa, MWCO). Three fractions with different molecular weights (>10 kDa, 5–10 kDa, and <5 kDa) were obtained. Peroxyl radical scavenging activities of these three separated fractions are shown in Table 2. The <5 kDa fraction possessed the highest peroxyl radical scavenging activity. IC_50_ (the half maximal inhibitory concentration) value of the <5 kDa fraction was 0.26 mg/mL, lower than that of the >10 kDa fraction (0.30 mg/mL) or the 5–10 kDa fraction (0.35 mg/mL). Accordingly, the <5 kDa fraction was further purified and separated using a Sephadex G-25 column. As shown in Figure 1A, four fractions were obtained. Their peroxyl radical scavenging activities were then determined. Fraction 3 (Fr. 3) exhibited the strongest peroxyl radical scavenging activity, with IC_50_ value of 0.12 mg/mL. Thus, Fr. 3 was further separated by RP-HPLC and five main fractions were obtained (Figure 1B). Fraction 3-3 (Fr. 3-3) showed the strongest peroxyl radical scavenging activity with an IC_50_ value of 0.028 mg/mL (Figure 1B), suggesting that Fr. 3-3 could possess potent antioxidant activity by scavenging peroxyl radicals. Thus, amino acid sequences of Fr. 3-3 was determined using Q-TOF ESI mass spectrometer. The purified peptide was identified as a tetrapeptide Trp-Asp-Val-Lys (named TAVL). The molecular weight of this tetrapeptide was 547.29 Da (Figure 1C). As shown in Table 3, the IC_50_ value of the TAVL was 51.16 μM for peroxyl radical scavenging activity, whereas positive control as ascorbic acid showed 19.26 μM of IC_50_ value, indicating the ascorbic acid is more potent scavenging activity. However, based on this results, we can confirm the superior antioxidant activity of TAVL.

### 2.3. Intracellular Antioxidant Activities of the Purified Peptide

**Figure 2:**
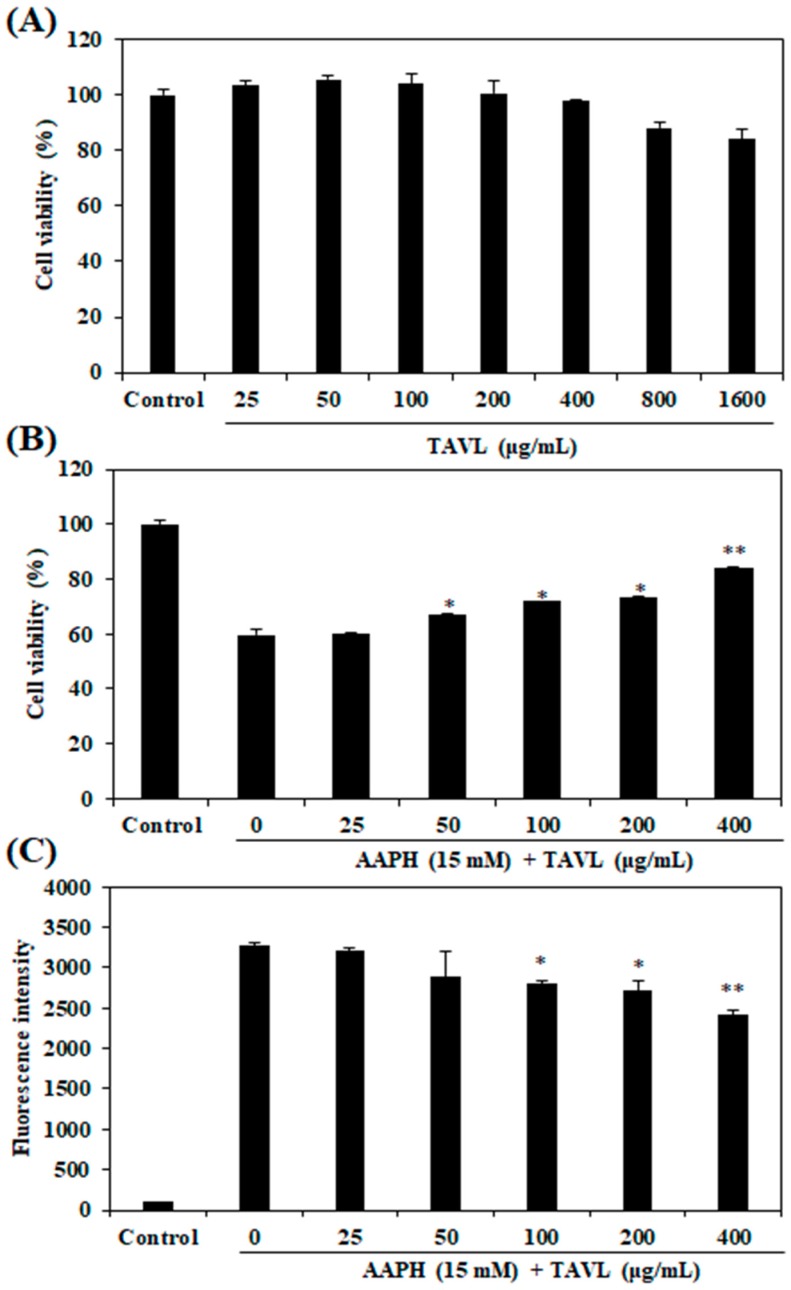
Protective effects of purified peptide (TAVL) against 2,2-Azobis-(2-amidinopropane) dihydrochloride (AAPH)-induced oxidative stress in Chang liver cells. Cells were treated with TAVL at indicated concentrations. (**A**) Cytotoxic effect of TAVL on viability of normal cells. After 24 of treatment with TAVL, cell viabilities were assessed by MTT assay. (**B**) Effect of TAVL on cell viability of AAPH-treated Chang liver cells. Cell viabilities were assessed by MTT assay. (**C**) Effect of TAVL on intracellular ROS generation in AAPH-treated Chang liver cells. Intracellular ROS generated was detected by 2′,7′-dichlorodihydrofluorescein diacetae (DCFH-DA) assay. Values are expressed as mean ± S.E. from triplicate experiments. Significant differences from only AAPH-treated group (positive control) were identified at * *p* < 0.05, ** *p* < 0.01 as analyzed by Duncan’s multiple range test. The control group represents the negative control that does not receive treatment of AAPH and sample in an experiment.

The cytotoxicity of the purified peptide (TAVL) was determined by 3-(4,5-Dimethylthiazol-2-yl)-2,5-diphenyltetrazolium bromide (MTT) assay at multiple TAVL concentrations (25, 50, 100, 200, 400, 800, and 1600 μg/mL) prior to evaluating its intracellular antioxidant activities. Results revealed that TAVL did not exhibit cytotoxicity at concentrations of up to 400 μg/mL, as compared with control survival (Figure 2A). Therefore, TAVL of non-toxic concentration was used to examine its protective effect against AAPH-induced cell damage in Chang liver cells. As shown in Figure 2B, AAPH treatment without TAVL significantly decreased cell viability. However, TAVL protected cells against cellular damage induced by AAPH in a dose-dependent manner. The generation of intracellular ROS could be measured by analyzing DCF fluorescence intensity levels. As Figure 2C shows, the fluorescence intensity of control group (AAPH and TAVL-untreated negative control) was recorded as 137, and the fluorescence intensity of only AAPH-treated cells was recorded as 3272. However, pretreatment of TAVL at 25, 50, 100, 200, and 400 μg/mL to cells mixed with AAPH reduced fluorescence intensities (intracellular ROS production levels) to 3202, 2899, 2806, 2719, and 2420, respectively. These results suggest that this antioxidant peptide (TAVL) could be developed into a potential bio-molecular candidate to inhibit cellular damage and intracellular ROS formation. Since this antioxidant peptide (TAVL) was found to exert antioxidant effects, its protective effect against AAPH-induced oxidative stress was further investigated using a zebrafish model in vivo.

### 2.4. Protective Effects of Antioxidant Peptide (TAVL) against AAPH-Induced Oxidative Stress in a Zebrafish Model In Vivo

**Figure 3:**
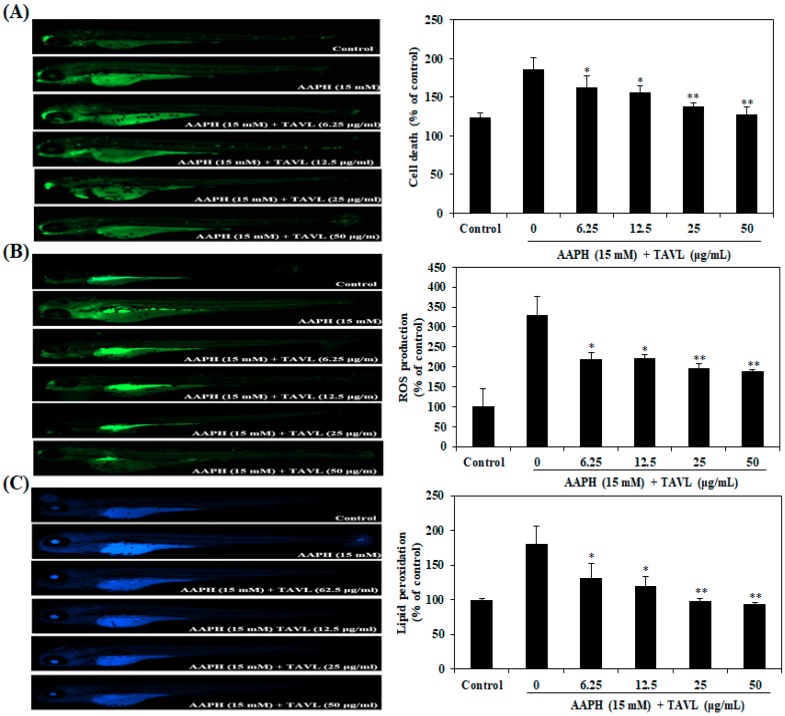
Protective effects of antioxidant peptide (TAVL) against AAPH-induced oxidative stress in zebrafish model. (**A**) Protective effect of TAVL on AAPH-induced cell death in zebrafish embryos. Cell death levels were measured after staining with acridine orange followed by image analysis and fluorescence microscopy. (**B**) Inhibitory effect of TAVL on AAPH-induced ROS production in zebrafish embryos. ROS levels were measured after staining with 2′,7′-dichlorodihydrofluorescein diacetae (DCF-DA) followed by image analysis and fluorescence microscopy. (**C**) Inhibitory effect of TAVL on AAPH-induced lipid peroxidation in zebrafish. Lipid peroxidation levels were by DPPP staining. The fluorescence intensity of individual zebrafish was quantified using Image J program. Values are expressed as mean ± S.E. Significant differences from only AAPH-treated group (positive control) were identified at * *p* < 0.05, ** *p* < 0.01 as analyzed by Duncan’s multiple range test. The control group represents the negative control that does not receive treatment of AAPH and sample in an experiment.

AAPH-induced oxidative stress could eventually lead to cell death and overproduction of ROS and lipid peroxidation. In the present study, the protective effects of TAVL against AAPH-induced cell death, ROS generation, and lipid peroxidation in the zebrafish were investigated. As shown in Figure 3A, cell death of zebrafish was significantly elevated by AAPH treatment compared to non-AAPH-treated zebrafish. However, cell death induced by AAPH in zebrafish was remarkably reduced by treatment with TAVL in a dose-dependent manner. Effects of TAVL on AAPH-induced ROS generation and lipid peroxidation level are shown in Figure 3B,C, respectively. The control, which contained no AAPH or TAVL, generated a clear image. After treatment with only AAPH, a fluorescence image was generated, suggesting that generation of ROS and lipid peroxidation had taken place in zebrafish embryos in the presence of AAPH. However, when zebrafish embryos were treated with TAVL prior to AAPH treatment, dose-dependent reductions in the generation of ROS and lipid peroxidation were observed. These results demonstrated that the antioxidant peptide (TAVL) could have a protective effect against oxidative stress through its antioxidant activity.

## 3. Discussion

Velvet antler is rich in proteins that may account for 60% (*w*/*w*) of dry matter [19]. However, velvet antler proteins have only received limited attention as a potential bioactive resource. Recently, the proteases have been successfully applied to extraction bioactive compounds from Velvet antler. Several studies have also reported that enzymatic hydrolysate extracted from velvet antler using protease show a variety of biological benefits, including anti-obesity, anti-inflammatory, and antioxidant effects [12,13,14,15]. However, there have been no reports about antioxidant peptides that can be extracted from velvet antler by enzymatic hydrolysis. Therefore, the aim of this study was to purify and identify antioxidant peptides from velvet antler enzymatic hydrolysates and to evaluate their antioxidant properties using peroxyl radicals scavenging assay. Protective effects of the purified peptide against AAPH-induced oxidative stress in Chang liver cells and in zebrafish model in vivo were also determined.

To obtain novel active antioxidant peptides, five commercial proteases were used under optimal conditions to hydrolyze velvet antler. Alcalase hydrolysate showed the highest peroxyl radical scavenging activity. In addition, several studies have suggested that Alcalase is useful for the production of bioactive peptide from food proteins [16,17,18,20,21]. Moreover, Alcalase can produce shorter peptide sequences and terminal amino acid sequences responsible for various bioactivities [16,18]. Thus, Alcalase hydrolysate was selected to identify antioxidant peptide for further studies.

The molecular weight of peptide is an important factor for its function [22]. Ultrafiltration (UF) is a simple and efficient technology for separating different molecular weights of molecules based on their molecular weights [23]. In this study, the Alcalase-proteolytic hydrolysate of velvet antler was separated to three fractions with different molecular weight (MW < 5 kDa, MW of 5–10 kDa, and MW > 10 kDa) by an UF system. Among these three MW groups, the <5 kDa fraction showed the strongest peroxyl radical scavenging activity. Previous reports have found that food proteins hydrolysates can be separated into three fractions (>10 kDa, 5–10 kDa and <5 kDa) by UF according to MW and that the <5 kDa fraction exhibits the strongest free radical scavenging activity [6,17,24]. Results of the present study also demonstrated that low MW fraction of Alcalase-proteolytic hydrolysate had higher free radical scavenging activity than higher MW fractions. Therefore, the <5 kDa fraction was selected for purification and identification of antioxidant peptide.

Sequential chromatography was used to purify antioxidant peptide from the active <5 kDa fraction, including Sephadex G-25 column gel filtration chromatography and RP-HPLC. After two-step isolation, we finally obtained the purified active peptide. Its amino acid sequence was determined with a Q-TOF ESI mass spectrometer. The purified antioxidant peptide was identified as a tetrapeptide Trp-Asp-Val-Lys (TAVL). The antioxidant peptide’s properties are based on its molecular weight, amino acid sequence, and composition [25]. As reported previously, peptides showing antioxidant activities with a lower molecular weight can more easily pass the intestinal barrier and exert biological effects [25,26,27]. The purified tetrapeptide Trp-Asp-Val-Lys in the present study had a low molecular weight of 547.29 Da. It showed good antioxidant activities in the present experiments, in agreement with previous reports. In addition, the composition of amino acids within sequences of the peptide is another important factor for its antioxidant effects [4]. Hydrophobic amino acids, including Trp, Pro, Tyr, Lys, Leu, Val, and His, play an important role in the radical scavenging effects of peptides [26]. In addition, it has been reported and proven that antioxidant peptides containing aromatic amino acid residues (Trp and Tyr) have strong antioxidative capacities because they can make active oxygen stable through direct electron transfer [1,4]. In this study, the identified tetrapeptide has three amino acid residues (Trp, Val, and Lys) responsible for it antioxidant activity. This represents 3/4 of its compositions.

Overproduction of ROS can induce oxidative stress, which can cause numerous diseases and disorders. Also, cellular damage by ROS-induced oxidative stress often impairs biomolecules function and leads to cell death [28]. AAPH is a free radical–generating compound widely used to mimic the oxidative stress state [24,29,30]. Hence, in order to assess the intracellular antioxidant activity of the purified antioxidant peptide, in this study, AAPH was used to induce oxidative stress. The level of ROS production in cells was detected via oxidant sensitive fluorescent probe DCFH-DA to measure whether purified antioxidant peptide could prevent AAPH-induced ROS generation and the resulting oxidative stressors. Our results showed that treatment of Chang liver cells with AAPH significantly increased intracellular ROS level. However, purified antioxidant peptide inhibited such ROS generation induced by AAPH. AAPH generates free radicals through reacting with oxygen, which resulting in rapid formation of peroxyl radicals. The presently demonstrated inhibitory action of purified antioxidant peptide on ROS production can be attributed to its peroxyl radical scavenging activity. This purified antioxidant peptide was evaluated further with regard to its protective effects against AAPH-induced cellar damage. Exposure of cells to AAPH resulted in a significant decrease of cell viability. However, treatment with the purified antioxidant peptide inhibited cell death, suggesting that the purified antioxidant peptide could protect cells against AAPH-induced cytotoxicity. These results suggest that the purified antioxidant peptide could have a protective effect against ROS-induced oxidative stress, thus leading to reduced cellular injuries.

Recent reports indicated that zebrafish can be used as a rapid and simple model to assess the antioxidant activity against oxidative stress in vivo [29,30]. Therefore, in the present study, we investigated the antioxidant effect of purified antioxidant peptide in vivo using the zebrafish model. In the current study, antioxidant effects of purified antioxidant peptide against AAPH-induced oxidative stress in zebrafish model were investigated. Our results showed that treating zebrafish embryos with AAPH-treatment significantly increased cell death and ROS levels. However, the purified antioxidant peptide inhibited such AAPH-induced cell death and ROS generation. Lipid peroxidation may be a form of free radical–caused cellular damage [31]. In the present study, lipid peroxidation significantly increased by AAPH treatment in zebrafish embryos. However, the purified antioxidant peptide inhibited such lipid peroxidation formation effectively. The protective effect of the purified antioxidant peptide against lipid peroxidation formation can be attributed to its antiperoxidative effect. Taken together, these results further support that the purified antioxidant peptide could be utilized as a natural antioxidant to potentially protect cells against oxidative stress.

In conclusion, an antioxidant tetrapeptide was purified and identified from Alcalase-proteolytic hydrolysate of velvet antler. The identified antioxidant peptide (Trp-Asp-Val-Lys, 547.29 Da) exhibited great antioxidant activity based on peroxyl radical scavenging assay. In addition, this tetrapeptide significantly inhibited AAPH-induced ROS production in Chang liver cells and in a zebrafish model in vivo. These results demonstrate that ROS reduction by tetrapeptide may contribute to attenuation of intracellular oxidative stress. This tetrapeptide could have potential applications in functional foods, nutraceutical, and pharmaceutical industries.

## 4. Materials and Methods

### 4.1. Chemicals and Reagents

2,2-Azobis-(2-amidinopropane) dihydrochloride (AAPH) and a-(4-pyridyl-1-oxide)-N-t-butylnitrone (4-POBN) were purchased from Sigma Chemical Co. (St. Louis, MO, USA). Protein proteases including pepsin, trypsin, and α-chymotrypsin were purchased from Sigma-Aldrich (St. Louis, MO, USA). Neutrase and Alcalase were purchased from Novozyme Co. (Novo Nordisk, Bagsvaerd, Denmark). Penicillin-streptomycin and trypsin-EDTA were purchased from Gibco-BRL (Burlington, ON, Canada). 2′,7′-dichlorodihydrofluorescein diacetae (DCFH-DA) and 3-(4,5-Dimethylthiazol-2-yl)-2,5-diphenyltetrazolium bromide (MTT) were obtained from Sigma-Aldrich (St. Louis, MO, USA). All other chemicals and reagents used were of analytical grade and obtained from commercial sources.

### 4.2. Sample Preparation

Velvet antler was obtained from a farmed elk Daesungsan Deer Farm (Daegwallyeong, Korea) at 75 days after casting. The fresh velvet antler was immediately sliced and lyophilized. The lyophilized velvet antler was ground into a fine powder and stored at −20 °C until use.

### 4.3. Preparation of Enzymatic Hydrolysates from Velvet Antler

Enzymatic hydrolysis was performed using five commercial proteases (trypsin, pepsin, α-chymotrypsin, Neutrase, and Alcalase) at their optimal conditions (pH and temperature) as described previously [24]. Briefly, one gram of dried velvet antler powder was added into 100 mL of distilled water. Each enzyme was then added to have a substrate to enzyme ratio of 100:1. Enzymatic hydrolysis was conducted under optimal conditions for 24 h, after which the hydrolysate was boiled at 100 °C for 10 min to inactivate the enzyme. These hydrolysates were clarified by centrifugation at 3000× *g* for 20 min to remove any unhydrolyzed residue. The supernatant of each hydrolysate was filtered, adjusted to pH 7.0, and stored for subsequent use in experiments.

### 4.4. Peroxyl Radical Scavenging Activity

Peroxyl radicals were generated by AAPH and their scavenging activities were measured using an electron spin resonance (ESR) spectrometer (JEOL, Tokyo, Japan) in accordance with the method described by Hiramoto et al. [32]. Briefly, 20 μL of 40 mM AAPH and 20 μL of 40 mM 4-POBN were mixed with 20 μL of PBS and 20 μL of indicated concentration of tested sample. The mixture solution was incubated at 37 °C in a water bath for 30 min and then transferred into a capillary tube. Experimental conditions were as follows: power, 10 mW; amplitude, 1 × 1000; modulation width, 0.2 mT; sweep width, 10 mT; sweep time, 30 s; and time constant, 0.03 s.

### 4.5. Isolation of Antioxidant Peptides from the Enzymatic Hydrolysate of Velvet Antler

#### 4.5.1. Fractionation According to the Molecular Weight

The enzymatic hydrolysate, which possess the highest peroxyl radical scavenging activity, was fractionated using the Millipore’s Lab scale TFF system (Millipore Corporation, Bedford, MA, USA) equated with ultrafiltration membranes (MWCO: 5 and 10 kDa) at 4 °C. Then, three fractions (>10 kDa, 5–10 kDa, and <5 kDa) were obtained.

#### 4.5.2. Purification of Antioxidant Peptides

The target fraction (500 mg) was loaded onto a Sephadex G-25 column (2.5 × 100 cm) pre-equilibrated with filtered distilled water. Elution was then carried out with filtered distilled water at a flow rate of 1.5 mL/min. Absorbance of each fraction at 220 nm was read and the sub-fractions were collected. The fraction with the highest peroxyl radical scavenging activity obtained was then subjected to reverse-phase high performance liquid chromatography (RP-HPLC) on an Atlantis T3 column (3 μm, 3.0 × 150 mm, Waters, NY, USA) with a linear gradient of acetonitrile (0–100% *v*/*v*, 30 min) at a flow rate of 1.0 mL/min. Elution peaks were detected at 220 nm.

#### 4.5.3. Identification of Purified Antioxidant Peptide

Molecular weight and amino acid sequences of antioxidant peptides purified from velvet antler were determined using a MicroQ-TOFIII mass spectrometer (Bruker Daltonics, Hamburg, Germany) coupled with electrospray ionization (ESI) source. The purified peptide was dissolved in distilled water and infused into the ESI source. Its molecular weight was determined by singly charged (M + H) state analysis in mass spectrum.

### 4.6. Experiments for Antioxidant Activity Assay Using Cells

#### 4.6.1. Cell Culture

The human hepatocyte–derived cell line termed Chang Liver were obtained from American Type Culture Collection (ATCC, Manassas, VA, USA) and it is well-known cell line used in various biological activities experiments including cellular antioxidant activity. Chang liver cells were cultured in Dulbecco’s modified Eagle’s medium (DMEM, Gibco-BRL, Burlington, ON, Canada) supplemented with 10% (*v*/*v*) heat-inactivated bovin serum (FBS, Gibco-BRL, Burlington, ON, Canada) and 1% (*v*/*v*) antibiotic. Cultures were maintained at 37 °C in a 5% CO_2_ incubator.

#### 4.6.2. Measuring Cytoprotective Effect by MTT Assay

Cytoprotective effect of the purified peptide was determined by a colorimetric MTT assay using Chang liver cells. Briefly, cells were seeded into a 96-well culture plates at cell density of 1 × 10^5^ cells/mL. After incubation for 16 h, cells were treated with various concentrations (25, 50, 100, 200, 400, 800, and 1600 μg/mL) of purified peptide. One hour later, 15 mM of AAPH was added to each well. Cells were then incubated for an additional 24 h at 37 °C. After incubation, 50 μL of MTT solution (stock concentration: 5 mg/mL in DPBS) was added into each well, and cells were incubated at 37 °C for 4 h. Supernatants were aspirated and formazan crystals in each well were dissolved in DMSO. Absorbance at 540 nm was then measured.

#### 4.6.3. Intracellular ROS Measurement

To detect levels of intracellular ROS, the DCFH-DA method was used as described previously [33]. Briefly, Chang liver cells were seeded into 96-well culture plates at cell density of 1 × 10^5^ cells/mL. After 16 h, cells were treated with various concentrations of purified peptide and then incubated at 37 °C. One hour later, 15 mM of AAPH was added to the culture. Cells were then incubated for an additional 30 min at 37 °C. DCFH-DA solution (5 μg/mL) was then introduced to cells. DCF-DA fluorescence was detected at an excitation wavelength of 485 nm and an emission wavelength of 535 nm using a Perkin-Elmer LS-5B spectrofluorometer.

### 4.7. In Vivo Zebrafish Model for Antioxidant Activity Assay

The adult zebrafish were maintained following our previous study [30,34]. At 7–9 h post-fertilization (hpf), zebrafish embryos were collected and arrayed in a 12-well plate (15 embryos/well) containing 2 mL embryo medium. The embryos were incubated with or without purified peptide for 1 h and then exposed to AAPH (15 mM) for 24 hpf. Thereafter, zebrafish embryos were transferred into fresh embryo medium and allowed to develop up to 72 hpf. Cell death, intracellular ROS, and lipid peroxidation in zebrafish were estimated according to previously reported methods [33,34]. Briefly, at 72 hpf, zebrafish embryos were transferred into 24-well plates and separately stained with specific fluorescent probe dyes to determine cell death (acridine orange), intracellular ROS (2′,7′-dichlorodihydrofluorescein diacetate (DCFH-DA)), and lipid peroxidation generation (diphenyl-1-pyrenylphosphine (DPPP). Following incubation for a specified period in the dye-containing media, embryos were rinsed with fresh embryo media, anesthetized, and then observed under a fluorescence microscope equipped with a CoolSNAP-Pro color digital camera (Olympus, Tokyo, Japan). The fluorescence intensities of individual zebrafish were quantified using Image J 1.46r software (Wayne Rasband, National Institutes of Health, Bethesda, MD, USA). Cell death, intracellular ROS, and lipid peroxidation generation were calculated by comparing fluorescence intensities of treated embryos to those of controls.

### 4.8. Statistical Analysis

Data are presented as means ± standard error (SE). Statistical comparisons of mean values were performed by analysis of variance (ANOVA) followed by a Duncan’s multiple range test using SPSS software.

## Figures and Tables

**Table 1:** Extraction yield and peroxyl radical scavenging activities of enzymatic hydrolysates from velvet antler.

Enzyme	Extraction Yield (%)	Radical Scavenging Activity (%) ^1)^
Trypsin	34.09	90.00 ± 1.69
Pepsin	12.39	78.58 ± 1.08
α-Chymotrypsin	38.96	89.54 ± 0.42
Neutrase	23.81	89.97 ± 1.05
Alcalase	29.75	93.78 ± 0.45

These values are expressed as mean ± S.E. from triplicate experiments. ^1)^ Radical scavenging activity was measured at 1 mg/mL by ESR spectrometry.

**Table 2:** Peroxyl radical scavenging activities of different molecular weight fractions from Alcalase hydrolysate of velvet antler.

Molecular Sizes	IC_50_ Value (mg/mL)
>10 kDa	0.30 ± 0.01 ^b^
5–10 kDa	0.35 ± 0.01 ^b^
<5 kDa	0.26 ± 0.02 ^a^

These values are expressed as mean ± S.E. from triplicate experiments. ^a,b^ Values with different alphabets are significantly different at *p < 0.05* as analyzed by Duncan’s multiple range test.

**Table 3:** Comparison with peroxyl radical scavenging activity by the tetrapeptide (TAVL) and ascorbic acid.

	IC_50_ Value (μM)
TAVL	51.16 ± 0.2
Ascorbic acid	19.26 ± 0.1

These values are expressed as mean ± S.E. from triplicate experiments.
